# Neuroprotective effects of GSK-343 in an in vivo model of MPTP-induced nigrostriatal degeneration

**DOI:** 10.1186/s12974-023-02842-6

**Published:** 2023-06-30

**Authors:** Deborah Mannino, Sarah Adriana Scuderi, Giovanna Casili, Valentina Bova, Laura Cucinotta, Marika Lanza, Alessia Filippone, Emanuela Esposito, Irene Paterniti

**Affiliations:** Department of Chemical, Biological, Pharmaceutical and Environmental Sciences, University of Messina, Viale Ferdinando Stagno D’Alcontres, 31, 98166 Messina, Italy

**Keywords:** Parkinson’s disease (PD), Epigenetic, Enhancer of zeste homolog 2 (EZH2), GSK-343, Neuroinflammation

## Abstract

Parkinson’s disease (PD) is characterized by the degeneration of dopaminergic nigrostriatal neurons, which causes disabling motor disorders. Scientific findings support the role of epigenetics mechanism in the development and progression of many neurodegenerative diseases, including PD. In this field, some studies highlighted an upregulation of Enhancer of zeste homolog 2 (EZH2) in the brains of PD patients, indicating the possible pathogenic role of this methyltransferase in PD. The aim of this study was to evaluate the neuroprotective effects of GSK-343, an EZH2 inhibitor, in an in vivo model of 1-methyl-4-phenyl-1,2,3,6-tetrahydropyridine (MPTP)-induced dopaminergic degeneration. Specifically, nigrostriatal degeneration was induced by MPTP intraperitoneal injection. GSK-343 was administered intraperitoneally daily at doses of 1 mg/kg, 5 mg/kg and 10 mg/kg, mice were killed 7 days after MPTP injection. Our results demonstrated that GSK-343 treatment significantly improved behavioral deficits and reduced the alteration of PD hallmarks. Furthermore, GSK-343 administration significantly attenuated the neuroinflammatory state through the modulation of canonical and non-canonical NF-κB/IκBα pathway as well as the cytokines expression and glia activation, also reducing the apoptosis process. In conclusion, the obtained results provide further evidence that epigenetic mechanisms play a pathogenic role in PD demonstrating that the inhibition of EZH2, mediated by GSK-343, could be considered a valuable pharmacological strategy for PD.

## Background

Parkinson’s disease (PD) is the most common neurodegenerative disorder [1] in which one risk factor is related to old age; in fact, the average age of onset is around 58–60 years [2]. However, other risk factors associated with PD are related to exposure to pesticides, toxic levels of manganese, trichlorethylene, carbon monoxide. In less than 10% of PD cases, the disease is associated to genetic mutations (familiar PD), such as the mutation of the α-synuclein (α-syn) gene that encodes for the α-syn protein. In the other 90% of the cases, the causes of the disease are unknown (idiopathic PD) [2]. The neuropathology of PD is characterized by the progressive and chronic degeneration of dopaminergic neurons in the substantia nigra pars compacta (SNpc). The SNpc plays important roles in the function of the basal ganglia including the control of voluntary movements. The loss of dopaminergic neurons causes subsequent depigmentation of the SNpc and accumulation of Lewy bodies (LBs) which are intraneuronal, round, eosinophilic inclusions with a hyaline nucleus that are composed of more than 90 proteins comprising α-syn [3]. α-syn has a propensity to become insoluble and form amyloid aggregates that alter the mitochondrial, lysosomal and proteasomal function with consequent damage to the biological membranes and alteration of the synaptic function that contribute to the degeneration of dopaminergic neurons [4]. Over the past two decades, inflammation in PD received great interest by many researchers, providing new insights into disease management. Inflammation can occur in response to molecules secreted by degenerating neurons thus contributing to the progression of PD. In the brains of patients with PD, astrogliosis and an increase in active microglial cells was observed not only in the nigrostriatal region, but also in various regions of the brain such as the hippocampus and cerebral cortex leading to the production of inflammatory mediators and oxygen reactive species (ROS). Numerous studies demonstrated increased levels of pro-inflammatory cytokines [5] which promote apoptotic cell death and subsequent phagocytosis of dopaminergic neurons suggesting that neuroinflammatory processes could represent a potent target for neuroprotection in PD [6]. Recent studies on epigenetic mechanisms have shown that DNA methylation and post-translational modifications of histone, necessary for gene expression in almost all tissues, are dysregulated in multiple neurodegenerative diseases and are closely involved in the processes of neuroinflammation [7]. For this reason, epigenetic mechanisms have become promising potential therapeutic targets for the treatment of neurodegenerative disorders such as Alzheimer’s disease (AD), PD, Huntington’s disease (HD) and amyotrophic lateral sclerosis (ALS) [8, 9]. In this context, great attention was given to the role of Enhancer of zeste homolog 2 (EZH2). EZH2 is a crucial catalytic subunit of the polycomb 2 (PRC2) repressive complex; it is a methyltransferase that acts on histone H3 lysine27 (H3K27) to produce trimethylated H3K27 (H3K27TM), involved in chromatin compaction and gene inactivation. Specifically, H3K27me3 inactivates the expression of suppressor of cytokine signaling 3 (Socs3) leading to the uncontrolled activity of NF-κB and increase of pro-inflammatory gene expression [10]. Recent studies have observed increased levels of H3K27me3 in the brains of patients with PD, indicating the possible pathogenic role of PRC2 in PD. These results indicated that EZH2-mediated dysregulation of histone H3K27me3 methylation altered gene expression (e.g., genes encoding α-synuclein) in SN neurons of PD patients [11]. An important study conducted by Penas et al. demonstrated that EZH2 and H3K27Me3 were co-localized in microglia, neurons and astrocytes, and EZH2 inhibition attenuates the expression of inflammation mediators at the brain level [12]. Therefore, considering the role of EZH2 in various pathological processes, several EZH2 inhibitors have been developed such as GSK-343. GSK-343 (1-isopropyl-1H-indazole-4-carboxamide) is a member of the class of indazoles with a potent and selective EZH2 inhibitor activity. Various in vitro and in vivo studies demonstrated the abilities of GSK-343 to modulate inflammation and apoptosis processes in several diseases such as colitis and also in cancer [13–16]; however, its effects in the context of neurodegenerative disease including PD, have not yet been investigated. Regarding pharmacokinetics, several studies have been conducted on EZH2 inhibitors, demonstrating their potential efficacy in crossing the blood brain barrier (BBB). Concerning GSK-343, little is known about its ability to cross the BBB, but despite this, Ratnam et al. [17] demonstrated that GSK126, an EZH2 inhibitor that has a similar molecular structure to GSK-343, can permeate into the intracranial tumor suggesting that it acts in the periphery and within the brain tumor, crossing the BBB. Furthermore, it has recently been shown that GSK-343 is able to inhibit glioma tumourigenesis and invasiveness by an in vivo orthotopic model of GBM, suggesting that it is able to cross BBB [18, 19]. Thus, considering the possible involvement of EZH2 in the progression of PD and in the processes of neuroinflammation, the aim of this study was to evaluate the effects of GSK-343, a selective EZH2 inhibitor, in a mouse model of 1-methyl-4-phenyl-1,2,3,6-tetrahydropyridine (MPTP)-induced dopaminergic neurodegeneration.

## Methods

### Materials

All compounds were obtained from Sigma-Aldrich Company Ltd. (Milan, Italy). All other chemicals were of the highest commercial grade available. All stock solutions were prepared in non-pyrogenic saline (0.9% NaCl; Baxter, Italy, UK).

### Animals

Adult male CD1 mice (30–35 g; 6–8 weeks old; Envigo, Italy) were housed in microisolator cages under pathogen-free conditions with 12 h light/12 h dark and provided with standard rodent chow and water. Animal experiments followed Italian regulations on the protection of animals used for experimental and other scientific purposes (DM 116,192) as well as EU regulations (OJ of EC L 358/1 12/18/1986) and the ARRIVE guidelines.

### MPTP-induced nigrostriatal degeneration

Animals received four intraperitoneal injections of 1-methyl-4-phenyl-1,2,3,6-tetrahydropyridine (MPTP) (20 mg/kg; Sigma-Aldrich, St. Louis, MO) dissolved in saline at 2-h intervals in 1 day as described by Campolo et al. [20]. The total dose for each mouse was 80 mg/kg. After 24 h, animals received GSK-343 by intraperitoneal administration at doses of 1, 5 and 10 mg/kg once daily for 7 consecutive days. Sham animals received vehicle only. Mice were killed 7 days after the first MPTP injection, and the brains were processed to perform several analyses.

### Experimental groups

Animals were randomly divided into the 8 following groups:

- Group 1: Sham + vehicle; vehicle solution (saline) was injected intraperitoneally for 7 consecutive days.
- Group 2: Sham + GSK-343 1 mg/kg; mice received intraperitoneally GSK-343 at dose of 1 mg/kg for 7 consecutive days starting 24 h after vehicle solution injection.
- Group 3: Sham + GSK-343 5 mg/kg; mice received intraperitoneally GSK-343 at dose of 5 mg/kg for 7 consecutive days starting 24 h after vehicle solution injection.
- Group 4: Sham + GSK-343 10 mg/kg; mice received intraperitoneally GSK-343 at dose of 10 mg/kg for 7 consecutive days starting 24 h after vehicle solution injection.
- Group 5: MPTP + vehicle: mice received intraperitoneally MPTP solution during the first day, and saline administration for 7 consecutive days starting 24 h after MPTP injection.
- Group 6: MPTP + GSK-343 1 mg/kg; mice received intraperitoneally GSK-343 at dose of 1 mg/kg for 7 consecutive days starting 24 h after MPTP injection.
- Group 7: MPTP + GSK-343 5 mg/kg; mice received intraperitoneally GSK-343 at dose of 5 mg/kg for 7 consecutive days starting 24 h after MPTP injection.
- Group 8: MPTP + GSK-343 10 mg/kg; mice received intraperitoneally GSK-343 at dose of 10 mg/kg for 7 consecutive days starting 24 h after MPTP injection.

The minimum number of mice for every technique was estimated with the statistical test “ANOVA: fixed effect, omnibus one-way” with the G-power software. This statistical test generated a sample size equal to *n* = 10 mice for each technique. The doses of GSK-343 were chosen according to a dose–response study performed in our laboratory. Experimental data regarding sham groups + GSK-343 1, 5 and 10 mg/kg, besides behavioral tests, were not shown because they did not result in either toxicity or improvement in comparison to sham group.

### Behavioral tests

Behavioral tests were performed 7 days after the last MPTP injection. The mice were placed in the behavior room for 5 min for 2 days for acclimation prior to the onset of behavioral testing [21].

### Pole test

The pole test was performed to evaluate movement disorders as previously described [22]. Mice were placed on top of a vertical pole, directed towards their cages. Under natural conditions, the mice will be oriented downwards along the length of the pole. The parameters evaluated were: time until the animal turned by 180°, called the turning time, and time until the animal dropped to the floor, total time.

### Elevated plus maze (EPM) test

Anxiety deficits were evaluated using elevated plus maze (EPM) system as described [23]. The elevated plus-maze test is one of the most used tests to measure anxiety-related behavior in rodent animals. The apparatus consists of two open arms, two closed arms and a center area. Mice were placed individually in the open arm, the time allowed to explore was 5 min. Behavioral representative of animal’s emotional state were: latency, frequency, and duration of visits in the open and closed arms. The total number of entries in the arms was used as a general index of activity. Data were analyzed by one-way analysis of variance, with the percentage of time spent in the closed arms as a percentage of the total.

### Immunohistochemical staining of tyrosine hydroxylase, anti-dopamine transporter and α-synuclein

Immunohistochemical staining was performed as previously described [24]. The brain sections of 7 µm were used for immunohistochemical staining. Sections were incubated overnight (O/N) with the following primary antibodies: anti-tyrosine hydroxylase (TH) (1:100; sc25269; Santa Cruz Biotechnology), anti-dopamine transporter (DAT) (1:100; sc-14002; Santa Cruz Biotechnology) and α-synuclein (α-syn; affinity purified rabbit polyclonal antibody raised against a peptide mapping at the C-terminus of α-synuclein of human origin) (1:100; sc-7011; Santa Cruz Biotechnology, Dallas, TX, USA). Then, the sections were washed with PBS and incubated with secondary antibody for 1 h. The reaction was revealed by a chromogenic substrate (brown DAB), and counterstaining with cresyl violet. As previously described by Impellizzeri et al., the analyses were performed by measuring the intensity of positive staining (brown staining) by computer-assisted color image analysis (Leica QWin V3, UK) [23]. The percentage area of immunoreactivity (determined by the number of positive pixels) was expressed as percent of total tissue area (cresyl violet staining). Photomicrographs were assessed densitometrically with Optilab software (Graftek, Mirmande, France) on a MacBook Pro computer (Apple, Cupertino, CA, USA). Analysis was carried out by assigning quantitative different criteria for staining intensity which included assignment of staining intensity using a scale of 0–10 (with 0 indicating a lack of brown immunoreactivity and 10 reflecting intense dark brown staining) by three different reliable expert observers. All stained sections were observed in a blinded manner. For immunohistochemistry, 20 × (50 µm scale bar) and 40 × (20 µm scale bar) were shown.

### Measurement of dopamine, 3,4‐dihydroxyphenylacetic acid (DOPAC), and homovanillic acid (HVA) levels in the striatum

Dopamine and its metabolites, DOPAC and HVA were quantified as previously shown [25]. Measurements were performed via high-performance liquid chromatography (HPLC) with electrochemical detection, using 0.15 M monochloroacetic acid, pH 3.0, and 200 mg/L sodium octyl sulfate, 0.1 mM EDTA, 4% acetonitrile, and 2 0.5% tetrahydrofuran as mobile phase. Data were collected and processed on a Dynamax (Rainin Instruments) computerized data manager.

### Luxol fast blue (LFB) staining

Luxol fast blue staining was used for evaluating myelin/myelinated axons and Nissl bodies according to manufacturer’s instructions (Bio-optica, cod: 04-200812). All stained sections were observed and analyed in a blinded manner. The magnifications 10 × (100 µm scale bar) and 20 × (50 µm scale bar) were shown.

### Western blot analysis

For western blot analysis, the ventral mesencephalon was isolated from brain tissues. Tissue samples were processed as previously reported by Campolo et al. [22]. The expression of EZH2, TRAF6, IĸB-α, NIK, COX2, iNOS, MMP2, MMP9, Bax, Bcl-2, and p53 were quantified in cytosolic fractions, meanwhile NF-ĸBp65 was quantified in the nuclear fraction. The following primary antibodies were used: anti-EZH2 (1:500, Invitrogen MA5-15101); anti-TRAF6 (1:500; Santa Cruz Biotechnology; Dallas, TX, USA sc-8409); anti-IĸBα (1:500; Santa Cruz Biotechnology; Dallas, TX, USA sc-1643), anti-NF-κB-inducing kinase (NIK) (1:500; Santa Cruz Biotechnology, Dallas, TX, USA; sc-6363); anti-inducible nitric oxide synthase (iNOS) (1:500; Santa Cruz Biotechnology, Dallas, TX, USA; sc-7271); anti-cyclooxygenase-2 (COX2) (1:500; Santa Cruz Biotechnology, Dallas, TX, USA; sc-376861); anti-MMP2 (1:500; Santa Cruz Biotechnology, Dallas, TX, USA sc-13595); anti-MMP9 (1:500; Santa Cruz Biotechnology, Dallas, TX, USA sc-13520); anti-Bcl-2 (1:500, Santa Cruz Biotechnology, Dallas, TX, USA; sc-7382), anti-Bax (1:500; Santa Cruz Biotechnology, Dallas, TX, USA; sc-7480), anti-p53 (1:500; Santa Cruz Biotechnology, Dallas, TX, USA; sc-126), and NF-κBp65 (1:500; Santa Cruz Biotechnology, Dallas, TX, USA; sc-8008). To confirm that the samples contained an equal protein concentration, membranes were incubated with primary anti-β-actin antibody (1:500; sc-47778; Santa Cruz Biotechnology, Dallas, TX, USA) for the cytosolic fraction or LAMIN A/C (1:500; sc-376248; Santa Cruz Biotechnology, Dallas, TX, USA) for the nuclear fraction. Signals were revealed by enhanced chemiluminescence (ECL) detection system reagent according to the manufacturer’s instructions (SuperSignal West Pico Chemiluminescent Substrate, Thermo Fisher Scientific, Waltham, MA, USA). The relative expression of the protein bands was quantified by densitometry with BIORAD ChemiDoc™XRS + software and standardized to β-actin or LAMIN A/C levels as internal control.

### Stereological quantitation of TH-positive neurons

Unbiased counting of TH^+^ dopaminergic neurons within substantia nigra par compacta (SNpc) was performed as previously described [22, 26]. The sections were incubated with polyclonal primary antibody mouse anti-TH (1:400, Santa Cruz Biotechnology) O/N. Brain sections were counterstained with cresyl violet, a Nissl stain. To count the number of TH + cells, StereoInvestigator software was used (Microbrightfield, Williston, VT). Cells were counted with a 10 × and 20 × objective, respectively, using a Leica DM2000 microscope (Leica, UK, EU). The area of interest for counting TH-immunoreactive cells was performed within a 50 × 50 × 5 µm frame on the same side of the brain, with an upper and lower control zone of 1 µm; for Nissl cell counting, the same sections were examined.

### ELISA kit for IL-1β, IL-2, IL-6, and IL-17

The levels of IL-1β, IL-2, IL-6, and IL-17A were evaluated on brain tissues extracts according to manufacturer’ instructions (Mouse IL-1 beta ELISA Kit, ab197742, Abcam; Mouse IL-2 ELISA kit, abx 050114, Abbexa; Mouse IL-6 ELISA Kit, ab100713, Abcam; Mouse IL-17A ELISA kit, EK0431, Boster).

### Immunofluorescence staining of GFAP and Iba-1

Brain sections were processed for immunofluorescence staining as previously described [27]. Sections were incubated with the following primary antibodies: anti-GFAP (1:100; sc-33673; Santa Cruz Biotechnology), anti-Iba-1 (1:100; sc-32725; Santa Cruz Biotechnology) in a humidified chamber O/N at 37 °C. After washes with PBS solution, sections were incubated with the secondary antibody, Alexa Fluor™ (1:1000 in PBS v/v, Molecular Probes, Altrincham, UK), Invitrogen, for 1 h at 37 °C. Subsequently, brain sections were washed in PBS and nuclear staining with 4′,6′-diamidino-2-phenylindole (DAPI; Hoechst, Frankfurt, Germany) (2 µg/mL) was added. Sections were observed and acquired at 40 × magnifications using a Leica DM2000 microscope (Leica, UK, EU). All stained sections were observed and analyed in a blinded manner.

### Statistical analysis

Experimental data are expressed as mean ± standard error of the mean (SEM) of N observations, in which N represents the number of animals studied. The results were examined by one-way ANOVA analysis of variance followed by a Bonferroni post hoc test for multiple comparisons. Only a *p*-value less than 0.05 was considered significant.

## Results

### GSK-343 treatment reduces MPTP-induced behavioral impairments

**Fig. 1:**
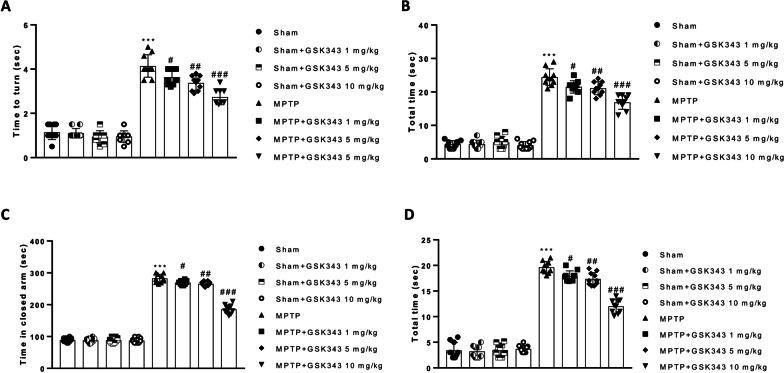
Effect of GSK-343 on behavioral impairments induced by MPTP. Mice subjected to MPTP injection showed a significant increase in behavioral deficits compared to the Sham group (**A–D**). Contrarily, GSK-343, at the two highest doses, considerably decreases “Time to turn” and “Time in closed arms” (**A–D**). Sham + GSK-343 administered groups were comparable to Sham mice (**A–D**). Data are representative of at least three independent experiments. Values are means ± SEM. One-way ANOVA test. ***p < 0.001 vs Sham; ^#^*p* < 0.05 vs MPTP; ^##^*p* < 0.01 vs MPTP; ^###^*p* < 0.001 vs MPTP

The pole test was performed in all experimental groups to assess MPTP-induced bradykinesia. This behavioral test showed that “time to turn” and “total time” were increased in mice injected with MPTP compared to the Sham group (Fig. 1A and B). Treatments with GSK-343 (1 mg/kg, 5 mg/kg, 10 mg/kg) showed a dose-dependent decrease in “Time to turn” and “Total time” compared to the MPTP group, thus suggesting a strong reduction in bradykinesia (Fig. 1A and B). The elevated plus-maze (EPM) test is one of the most used tests to measure anxiety-related behavior in rodent animals. EPM test reported an increase of time spent in the closed arm and total time, by MPTP-intoxicated mice compared to the Sham group (Fig. 1C and D). Instead, GSK-343 treatments at the dose-dependent manner reduced the time spent with closed arms compared to MPTP mice (Fig. 1C and D).

### GSK-343 administration reduced loss of tyrosine hydroxylase (TH) expression following MPTP injection

**Fig. 2:**
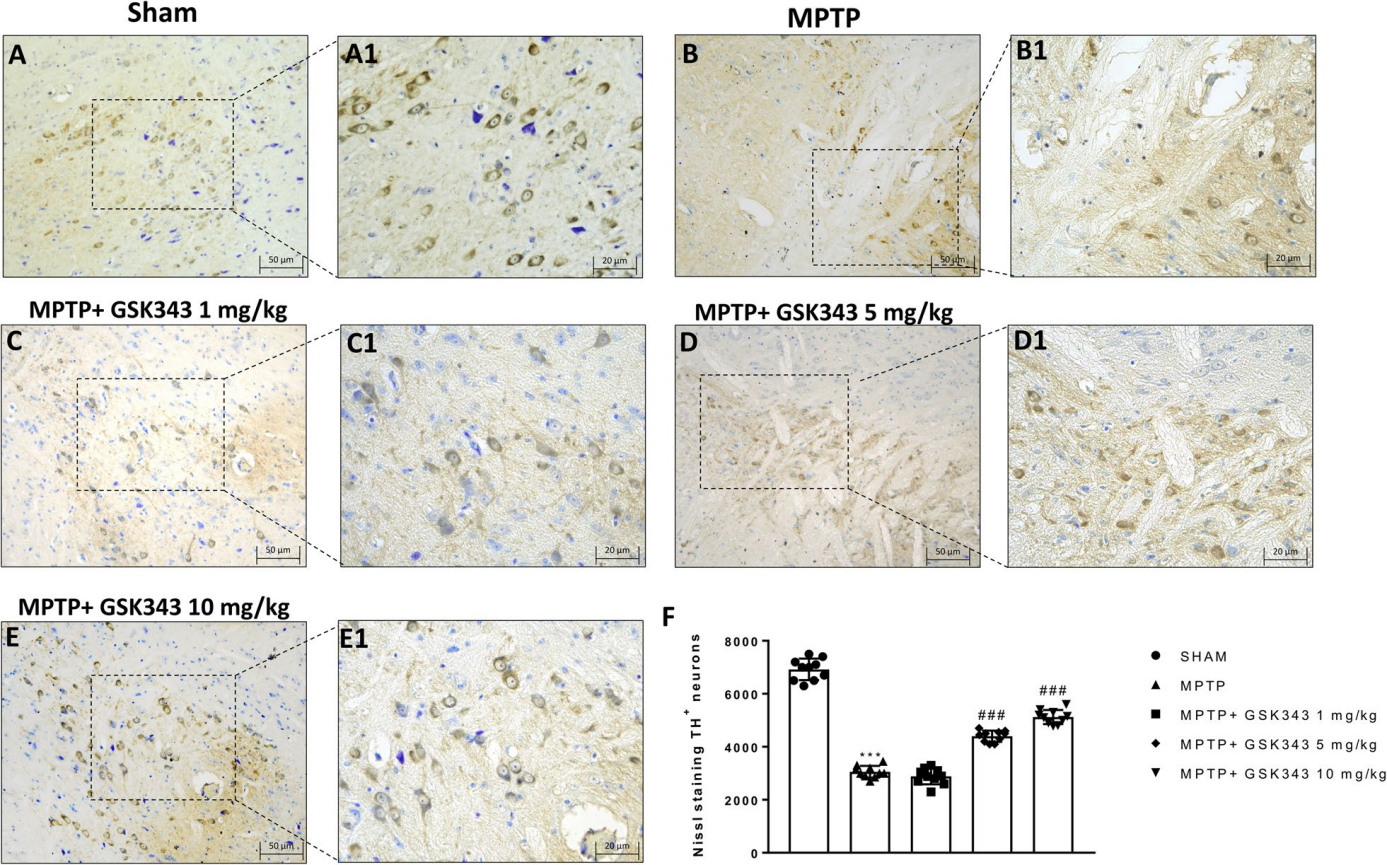
Effect of GSK-343 treatment on TH expression. MPTP mice exhibited an extensive loss of TH-positive neurons (**B, B1**, score **F**), compared to the Sham group (**A, A1**, score **F**). GSK-343 1 mg/kg did not reduce the loss TH-positive neurons (**C, C1**, score **F**). GSK-343 5 mg/kg treatment, and especially at the dose of 10 mg/kg, restored the numbers of TH + neurons (**D, D1**; **E, E1**, score **F**). Data are representative of at least three independent experiments. Values are means ± SEM. One-way ANOVA test. ***p < 0.001 vs Sham; ^###^*p* < 0.001 vs MPTP

TH is the enzyme that catalyzes the conversion of L-tyrosine to dihydroxyphenylalanine (DOPA), a precursor of dopamine. In this context, Parkinson’s disease can be considered as a TH deficiency syndrome of the striatum [28]. Our study investigated if GSK-343 could protect against MPTP-induced loss of TH-positive dopaminergic neurons in striatal by TH immunoreactivity staining of midbrain sections. Eight days after MPTP intoxication, we observed a substantial loss in the number of TH-positive neurons in MPTP-injected mice (Fig. 2B and B1, score panel F) compared to sham mice (Fig. 2A and A1, score panel F). Administration of GSK-343 at a dose of 1 mg/kg did not reduce the loss TH-positive neurons induced by MPTP intoxication (Fig. 2C and C1, score panel F) demonstrating ineffective. On the contrary, treatment with GSK-343 at the dose of 5 mg/kg (Fig. 2D and D1, score panel F), and mainly at 10 mg/kg (Fig. 2E and E1, score panel F), significantly preserved the number of TH-positive neurons compared to MPTP-injected midbrain sections.

### GSK-343 prevented dopamine transporter and its metabolites depletion after MPTP intoxication

**Fig. 3:**
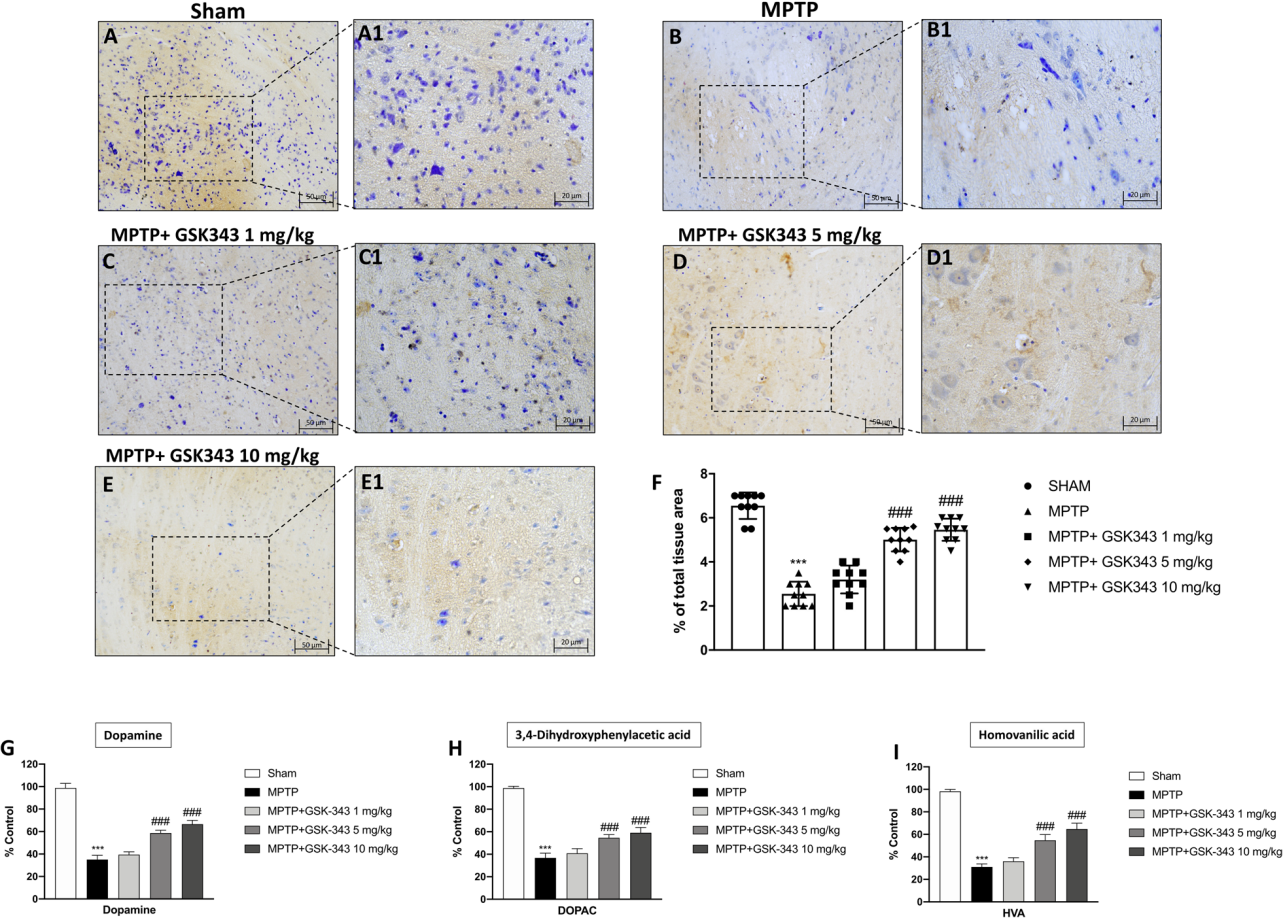
Effect of GSK-343 on dopamine pathway. MPTP mice showed a significant decrease of DAT expression in substantia nigra (**B, B1**, score **F**) compared to the Sham group (**A, A1**, score **F**). GSK-343 administration, at the doses of 5 mg/kg (**D, D1**, score **F**) and more effectively at the dose of 10 mg/kg (**E, E1**, score **F**) increased DAT-positive cells compared to MPTP mice. GSK-343 1 mg/kg did not demonstrate significant neuroprotection (**C, C1**, score **F**). MPTP-injected animals exhibited a considerable loss of dopamine and its metabolites, compared to the Sham mice; contrarily, treatment with GSK-343 at the doses of 5 mg/kg and 10 mg/kg increased metabolites levels (**G–I**). Data are representative of at least three independent experiments. Values are means ± SEM. One-way ANOVA test. ***p < 0.001 vs Sham; ###*p* < 0.001 vs MPTP

To investigate the protective effects of GSK-343 treatment on the dopamine pathway, we evaluated DAT expression in the substantia by immunohistochemistry analysis. The obtained results demonstrated a significant loss of DAT-positive staining in MPTP-injected mice (Fig. 3B, B1, F score) compared to the Sham group (Fig. 3A, A1, F score). Recovery of DAT levels was notable after administration of GSK-343 at the 5 mg/kg dose (Fig. 3D, D1, F score) and more effectively at the 10 mg/kg dose (Fig. 3E, E1, F score), compared to the MPTP group. Conversely, the lowest dose of GSK-343 (1 mg/kg) did not significantly improve DAT expression (Fig. 3C, C1, F score). In addition, we confirmed the neuroprotective role of GSK-343 by evaluating striatal levels of dopamine and its metabolites: DOPAC and HVA. MPTP intoxication significantly reduced levels of striatal dopamine, DOPAC and HVA compared with sham mice. Treatment with GSK-343 1 mg/kg did not prevent this depletion, while GSK-343 at a dose of 5 mg/kg, and especially 10 mg/kg, showed a significant restoration of dopamine and its metabolite levels.

### Protective effects of GSK-343 on α-syn-induced neurodegeneration

**Fig. 4:**
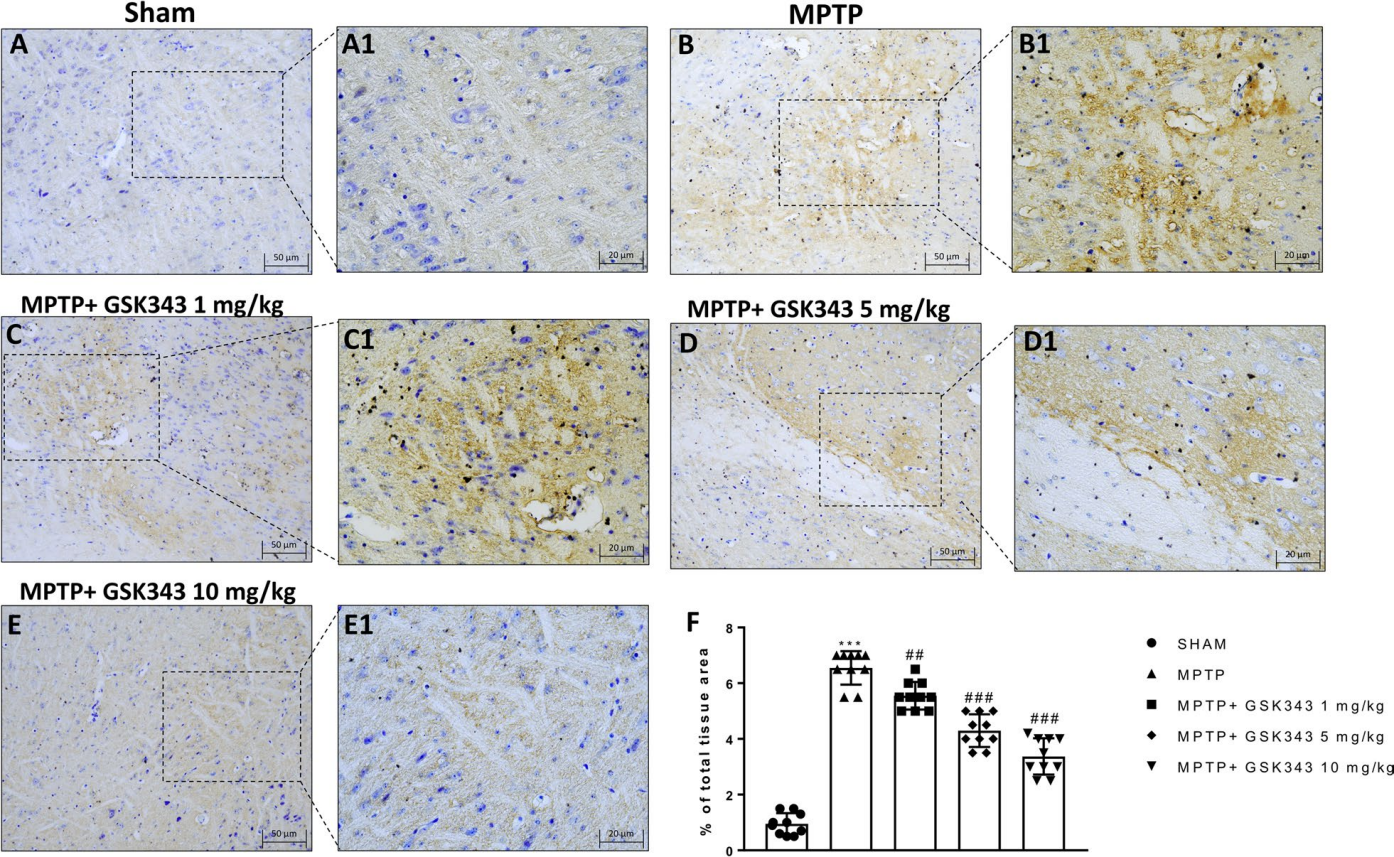
GSK-343 treatment preserved α-Syn accumulation. MPTP-injected mice showed an increase in the number of α-syn aggregates in substantia (**B, B1**, score **F**), compared to the Sham group (**A, A1**, score **F**). GSK-343 treatment at doses of 1 mg/kg, 5 mg/kg and 10 mg/kg revealed a reduction in the number of α-syn aggregates (**C, C1, D, D1** and **E, E1**, score **F**). Data are representative of at least three independent experiments. Values are means ± SEM. One-way ANOVA test. ***p < 0.001 vs Sham; ^##^*p* < 0.01 vs MPTP; ^###^*p* < 0.001 vs MPTP

Since the accumulation of α-syn in the dopaminergic neurons of the substantia nigra is a critical indicator of PD [29], we wanted to evaluate the expression of this protein in order to determine the ability of GSK-343 to counteract the neurodegenerative process. Immunohistochemical analysis of midbrain sections demonstrated a significant accumulation of α-syn in MPTP-injured mice (Fig. 4B and B1, score panel F) compared with sham midbrain sections which showed basal levels of α-syn (Fig. 4A and A1, score panel F). Furthermore, our results showed that even at the lowest dose, GSK-343 (1 mg/kg) was able to significantly counteract synuclein aggregates compared to the MPTP-injected mice (Fig. 4C and C1, score panel F). Increasing the dosage of GSK-343 (5 mg/kg and 10 mg/kg), a greater reduction of α-syn deposition was observed (Fig. 4D, D1 and E, E1, score panel F).

### GSK-343 treatment reduces myelin degradation in dopaminergic neurons

**Fig. 5:**
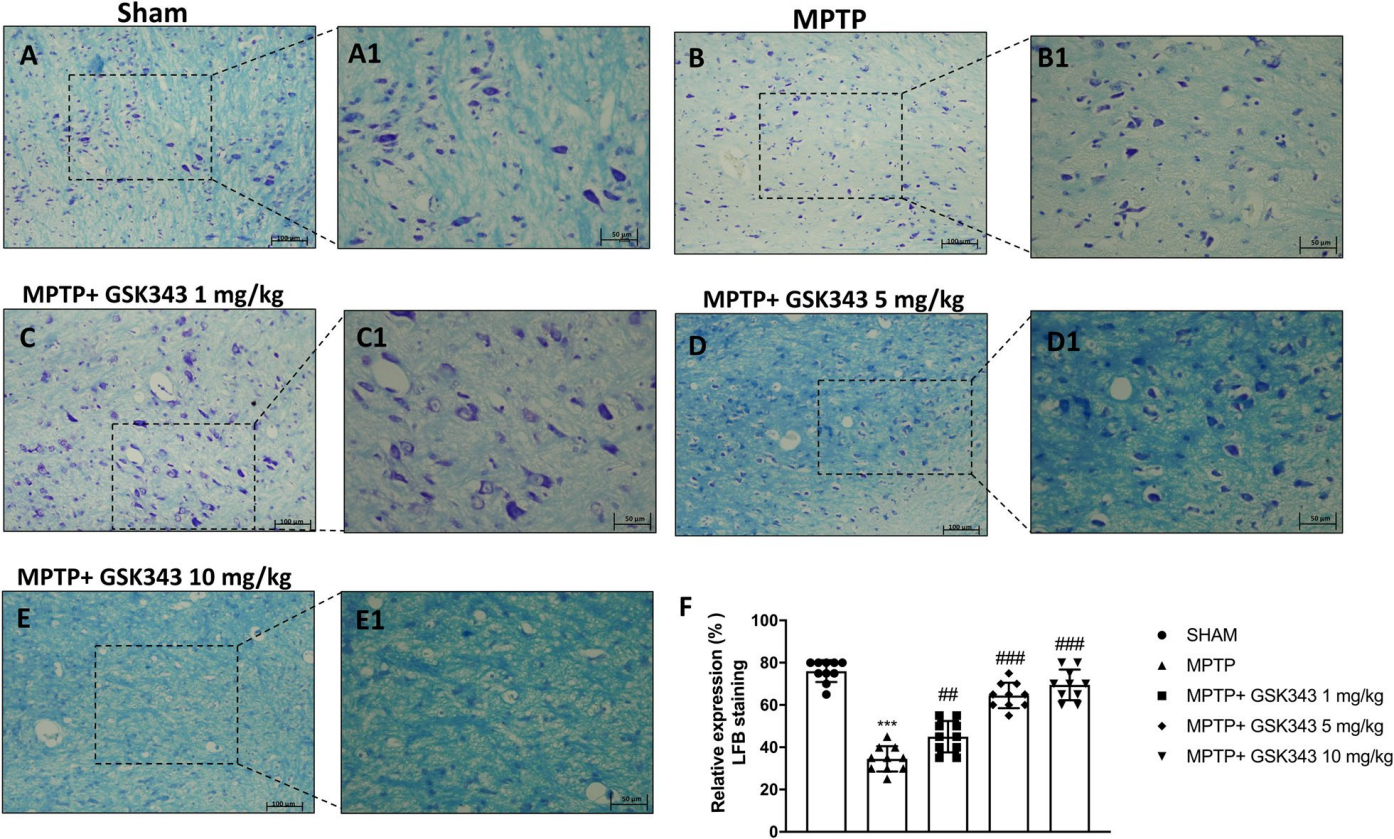
Effects of GSK-343 on the myelination process. LFB staining showed a significant loss of myelin in MPTP-injected mice (**B, B1**; score **F**) compared to Sham mice (**A, A1**; score **F**). Treatment with GSK-343 significantly restored the myelin presence (**C, C1, D, D1, E, E1**; score **F**). Data are representative of at least three independent experiments. Values are means ± SEM. One-way ANOVA test. ***p < 0.001 vs Sham; ^##^*p* < 0.01 vs MPTP; ^###^*p* < 0.001 vs MPTP

Alteration in myelin is a key pathological feature of neurodegenerative diseases. Myelin sheath is essential for numerous neuronal processes such as impulse transmission, synaptic plasticity, and blood brain barrier integrity [30]. For the evaluation of the myelin structure and the severity of the dopaminergic neurons alteration, Luxol fast blue staining was performed. Our data showed that in sham animals the myelin structure was clearly stained by luxol quickly. In contrast, eight days after MPTP injury, significant myelin loss was observed in MPTP-injected mice compared to sham mice (Fig. 5A, A1 and B, B1, score panel F). Myelin degradation was reduced in a dose-dependent manner in GSK-343 treated mice. Treatment with GSK-343 at a dose of 1 mg/kg significantly restored the presence of myelin compared to MPTP-injected mice (Fig. 5C, C1, score panel F), but a greater increase in myelin recovery was observed at higher doses of 5 mg/kg and 10 mg/kg of GSK-343 (Fig. 5D, D1 and E, E1, score panel F).

### Effect of GSK-343 on EZH2/TRAF6/NF-κB pathway

**Fig. 6:**
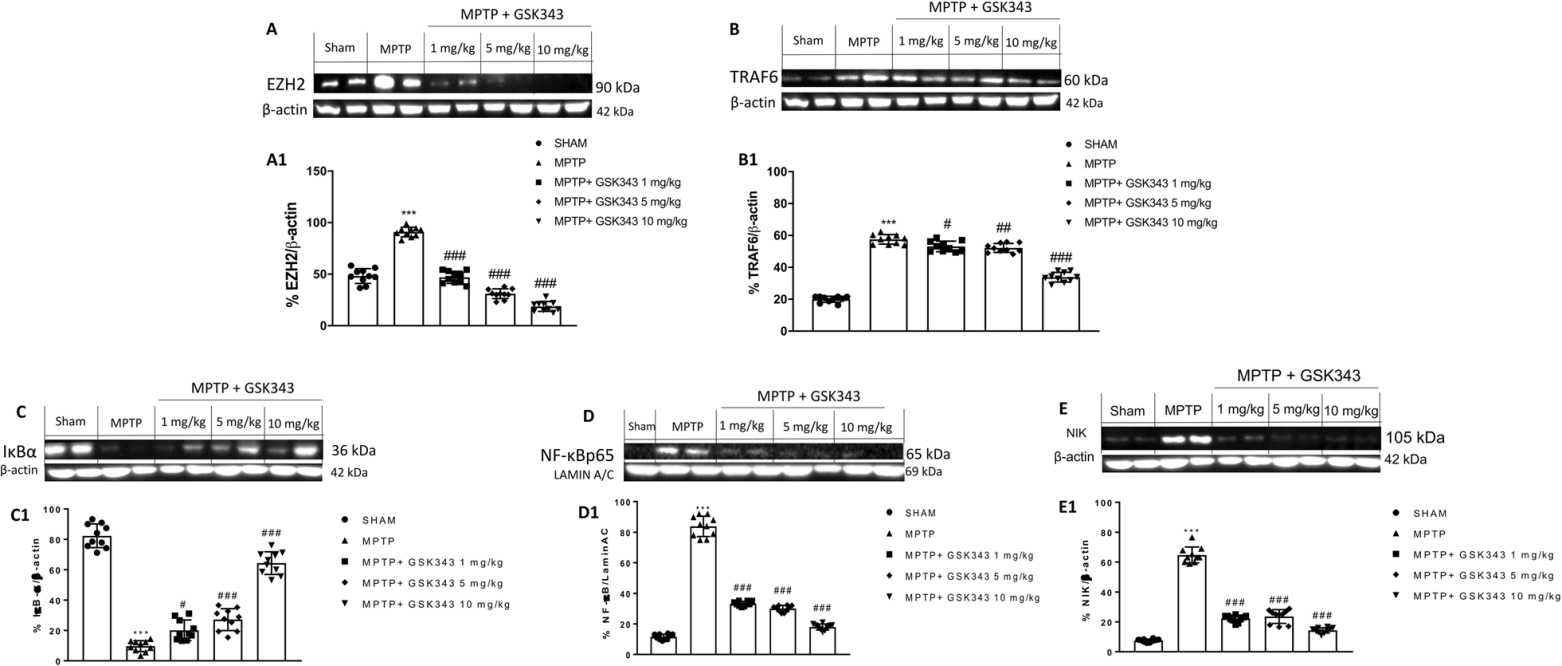
GSK-343 administration modulated neuroinflammation after MPTP injection. Western blot analysis revealed an increase of EZH2 and TRAF6 expression in MPTP-injected mice, compared to the Sham mice however, GSK-343 (1 mg/kg, 5 mg/kg and 10 mg/kg) administration was able to reduce their levels (**A**, **B**, densitometric analysis **A1–B1a**). MPTP-injected mice showed a decrease expression of IĸB-α, compared to the Sham mice (**C**, densitometric analysis **C1**). NF-kB and NIK levels resulted elevated in the MPTP-intoxicated mice compared to Sham animals (**D, E** densitometric analysis **D1–E1**). GSK-343 (1 mg/kg, 5 mg/kg and 10 mg/kg) administration increased IKB-α levels and at the same time reduced NF-kB and NIK expression. Data are representative of at least three independent experiments. Values are means ± SEM. One-way ANOVA test. ***p < 0.001 vs Sham; ^#^*p* < 0.05 vs MPTP; ^###^*p* < 0.001 vs MPTP

Western blot analysis was performed to detect EZH2 protein levels (Fig. 6A, see densitometric analysis A1). Results showed that EZH2 expressions increased in MPTP mice compared to baseline levels of sham mice. As expected, the selective antagonist, GSK-343, downregulated EZH2 at all administered doses. Our results demonstrated that GSK-343 administration (especially at the highest doses of 5 mg/kg and 10 mg/kg) significantly reduced TRAF6 expression compared to MPTP-injected mice (Fig. 6B, see densitometric analysis B1). Moreover, our data showed that IκBα degradation was markedly diminished in MPTP-injured mice compared to sham mice, whereas GSK-343 treatment acted to maintain IκBα cytosolic activity in a dose-dependent manner (Fig. 6C, see densitometric analysis C1). Conversely, in MPTP-injected mice there was a significantly increased of nuclear NF-κB expression compared to sham mice, while, GSK-343 treatment at the doses of 1 mg/kg, 5 mg/kg and 10 mg/kg significantly attenuated NF-κB nuclear translocation when compared to MPTP-injured mice (Fig. 6D, see densitometric analysis D1). Non-canonical NF-κB pathway is involved in MPTP-induced DA damage [31], and leads to an increase in the cytosolic activity of NIK, a kinase protein which phosphorylates and activates IKKα with subsequent activation of NF-κB. In this context, we evaluated an increased NIK levels in MPTP-injected mice, compared to sham mice; however, this increase was considerably reduced by GSK-343 treatment at the doses of 1 mg/kg, 5 mg/kg and 10 mg/kg compared to MPTP-injected mice (Fig. 6E, see densitometric analysis E1).

### GSK-343 treatment reduced cytokine levels induced by MPTP intoxication

**Fig. 7:**
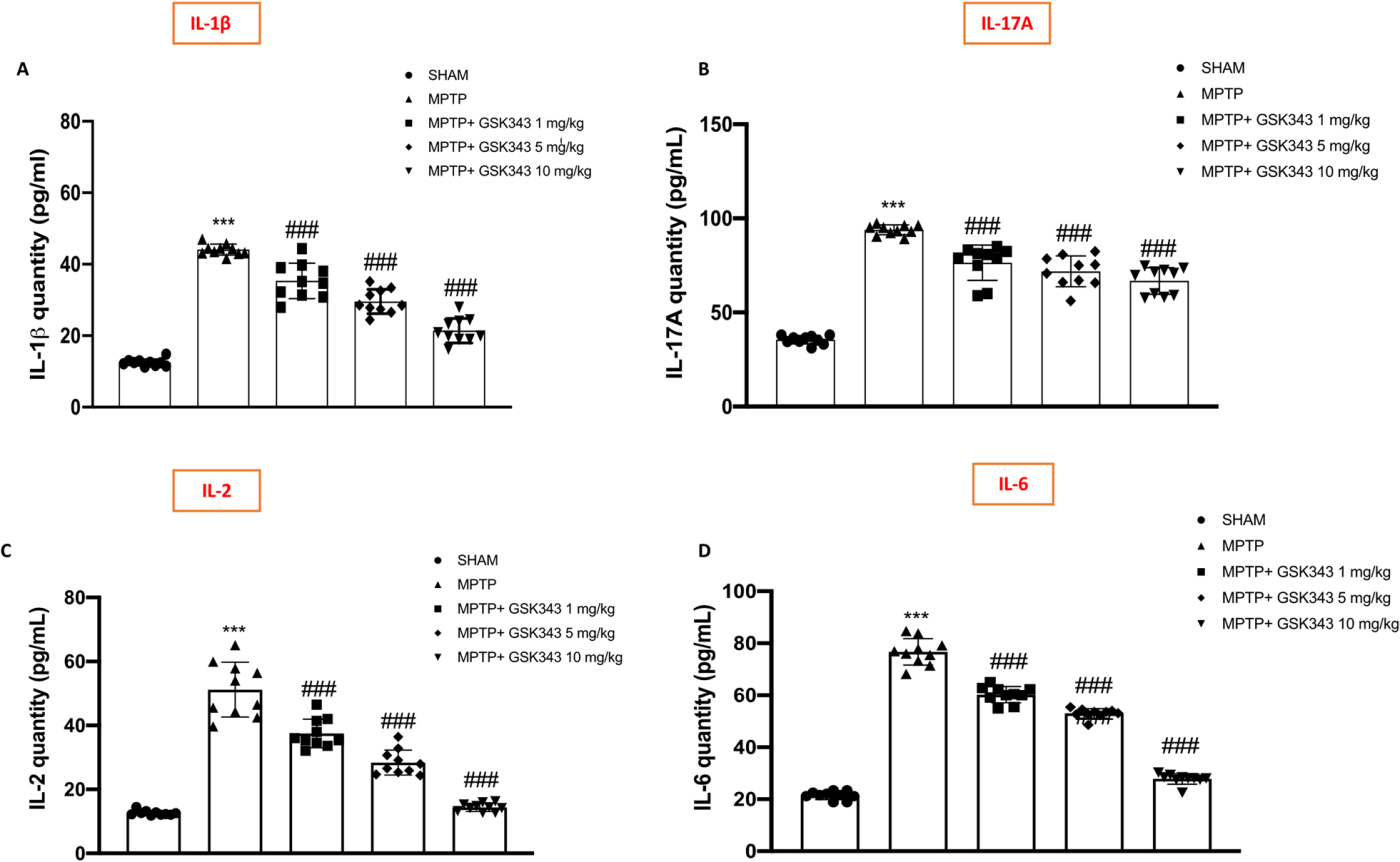
Effect of GSK-343 treatments on pro-inflammatory cytokine. ELISA assay revealed a significant upregulation of pro-inflammatory cytokines expression such as IL-1β, IL-6, IL-2 and IL-17A in the MPTP mice, compared to the Sham mice (**A–D**). These expressions were considerably reduced following GSK-343 treatments at all doses (1 mg/kg, 5 mg/kg and 10 mg/kg). Data are representative of at least three independent experiments. Values are means ± SEM. One-way ANOVA test. ***p < 0.001 vs Sham; ^###^*p* < 0.001 vs MPTP

Activation of NF-κB promotes transcription of pro-inflammatory genes resulting in increased levels of pro-inflammatory cytokines which contribute to the inflammatory process [32]. For this purpose, we evaluated the expression of pro-inflammatory cytokines like IL-1β, IL-6, IL-2 and IL-17A by ELISA assay. Our data showed a significant upregulation of IL-1β, IL-6, IL-2 and IL-17A in MPTP-intoxicated mice compared to the Sham group; however, GSK-343 (1 mg/kg, 5 mg/kg and 10 mg/kg) administration demonstrated its ability to significantly reduce cytokine levels in a dose-dependent manner, compared to MPTP-injected mice (Fig. 7A–D).

### GSK-343 treatment counteracts astrogliosis and microgliosis

**Fig. 8:**
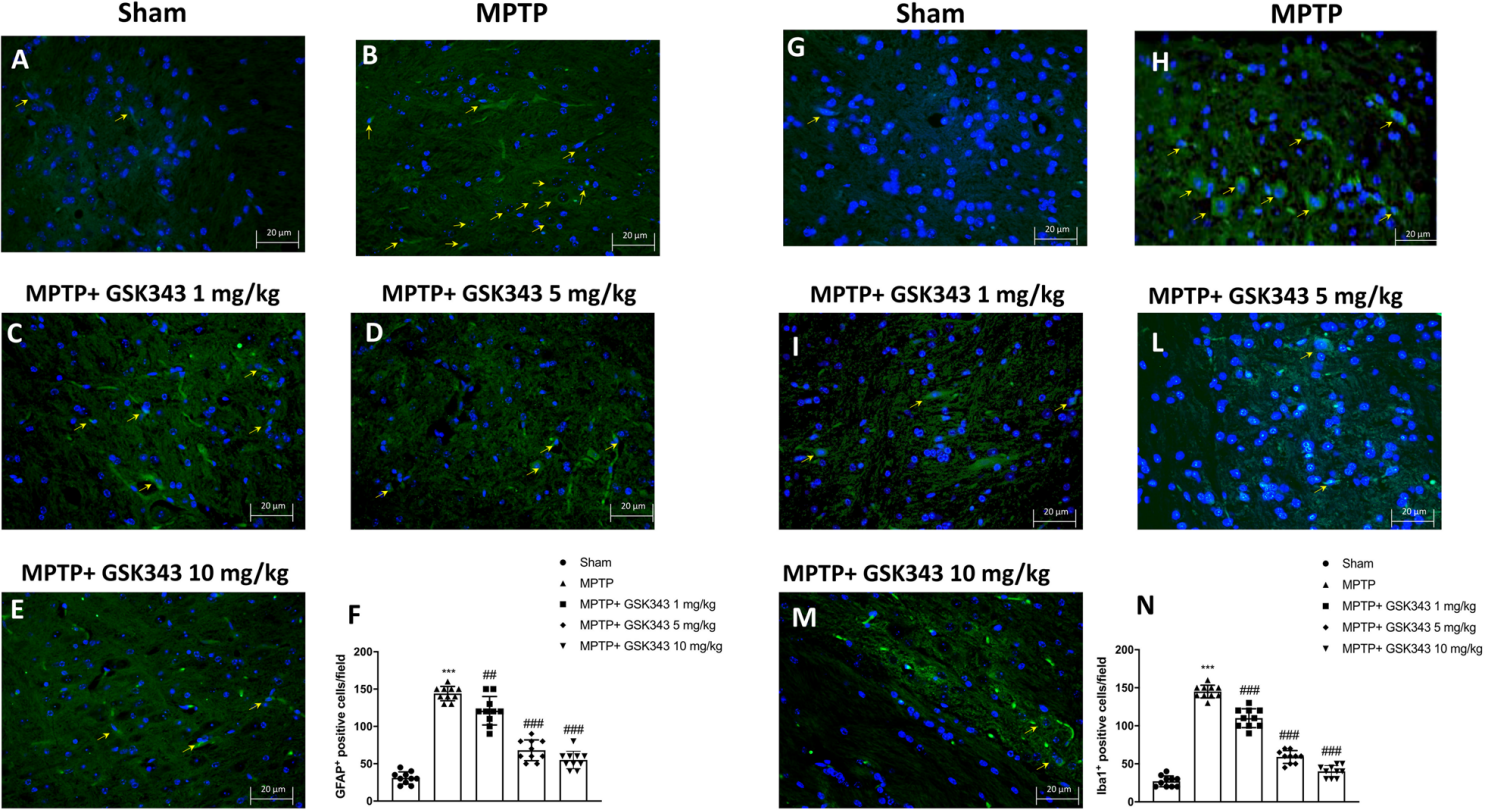
Immunofluorescence analysis of Iba1 and GFAP after MPTP intoxication. Tissues sections were stained with antibodies against Iba1 (green) or GFAP (green) and DAPI to highlight cell nuclei (blue). MPTP-injected mice showed high number of GFAP + cells (**B**, score **F**) and IBA-1 + cells (**H**, score **N**), compared to the Sham groups (**A**, score **F**; **G**, score **N**). Number of GFAP and IBA-1 + cells decreased after GSK-343 treatment at the dose of 1 mg/kg (**C**, score **F**; **I**, score **N**) more effectively at the highest dose of 5 mg/kg and 10 mg/kg (**D, E**, score **F**; **L–M**, score **N**). Data are representative of at least three independent experiments. Values are means ± SEM. One-way ANOVA test. ***p < 0.001 vs Sham; ^##^*p* < 0.01 vs MPTP; ^###^*p* < 0.001 vs MPTP

Reactive astrogliosis and microglia activation are two mechanisms that contribute to dopaminergic neuronal loss and neuroinflammation in Parkinson’s disease [33]. Using immunofluorescence analysis, we studied the expression of GFAP and Iba-1, known markers of astrocytosis and microgliosis, respectively. Our results demonstrated that after MPTP injection, there was a significant increase of both IBA-1 and GFAP-positive cells (Fig. 8B and H; score panel F and N) compared to sham mice (Fig. 8A and G; score panel F and N). Differently, administration of GSK-343 significantly reduced the number of both IBA-1 and GFAP-positive cells in a dose-dependent manner compared to MPTP-injected mice (Fig. 8C–E, score panel F for GFAP and panel I, L, M, score panel N for Iba1).

### GSK-343 treatment reduced COX-2, iNOS and MMPs expression

**Fig. 9:**
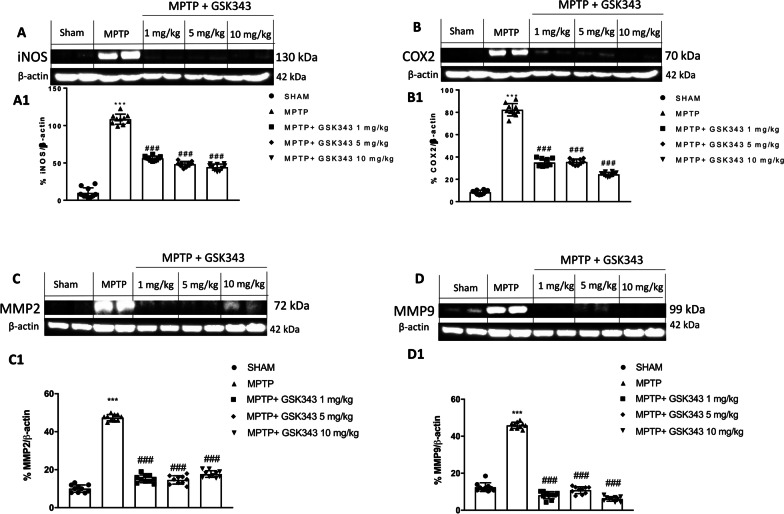
Effect of GSK-343 on pro-inflammatory enzymes and MMPs. Representative blots of iNOS, COX-2 revealed a significant upregulation of these markers in MPTP-injected mice compared to Sham mice. These expressions were considerably reduced following GSK-343 treatment (**A, B**; densitometric analysis **A1–B1**). MMP2 and MMP9 levels were increased in MPTP-intoxicated mice, while GSK-343 administration was able to reduce their levels (**C, D**; densitometric analysis **C1, D1**). Data are representative of at least three independent experiments. Values are means ± SEM. One-way ANOVA test. ***p < 0.001 vs Sham; ^###^*p* < 0.001 vs MPTP

Overexpression of COX-2 and iNOS plays an important role in inducing neuroinflammation during the pathogenesis of PD. In this context, our study demonstrated that expression of pro-inflammatory enzymes iNOS and COX-2 was significantly elevated in MPTP-injected mice in comparison to sham mice. GSK-343 treatment at the doses of 1 mg/kg, 5 mg/kg and 10 mg/kg was able to significantly reduce both iNOS and COX-2 expression compared MPTP-injected mice (Fig. 9A and B, see densitometric analysis A1 and B1). The increase of pro-inflammatory enzymes, such as iNOS and COX-2, and oxidative stress is closely related to the release of matrix metalloproteinases (MMPs). In particular, MMP-2 and MMP-9 are released from apoptotic dopaminergic neurons contributing to the inflammatory process [34]. Therefore, we evaluated whether GSK-343 could reduce the expression of MMP-2 and MMP-9. Western blot analysis showed that MPTP administration significantly increased the expression of MMP-2 and MMP-9 compared to sham mice. Interestingly, GSK-343 administration (1 mg/kg, 5 mg/kg and 10 mg/kg) reduced MMP-mediated inflammation by attenuating the activities of MMP-2 and MMP-9 (Fig. 9C and D, see densitometric analysis C1 and D1).

### Treatment with GSK-343 attenuates the degree of apoptosis

**Fig. 10:**
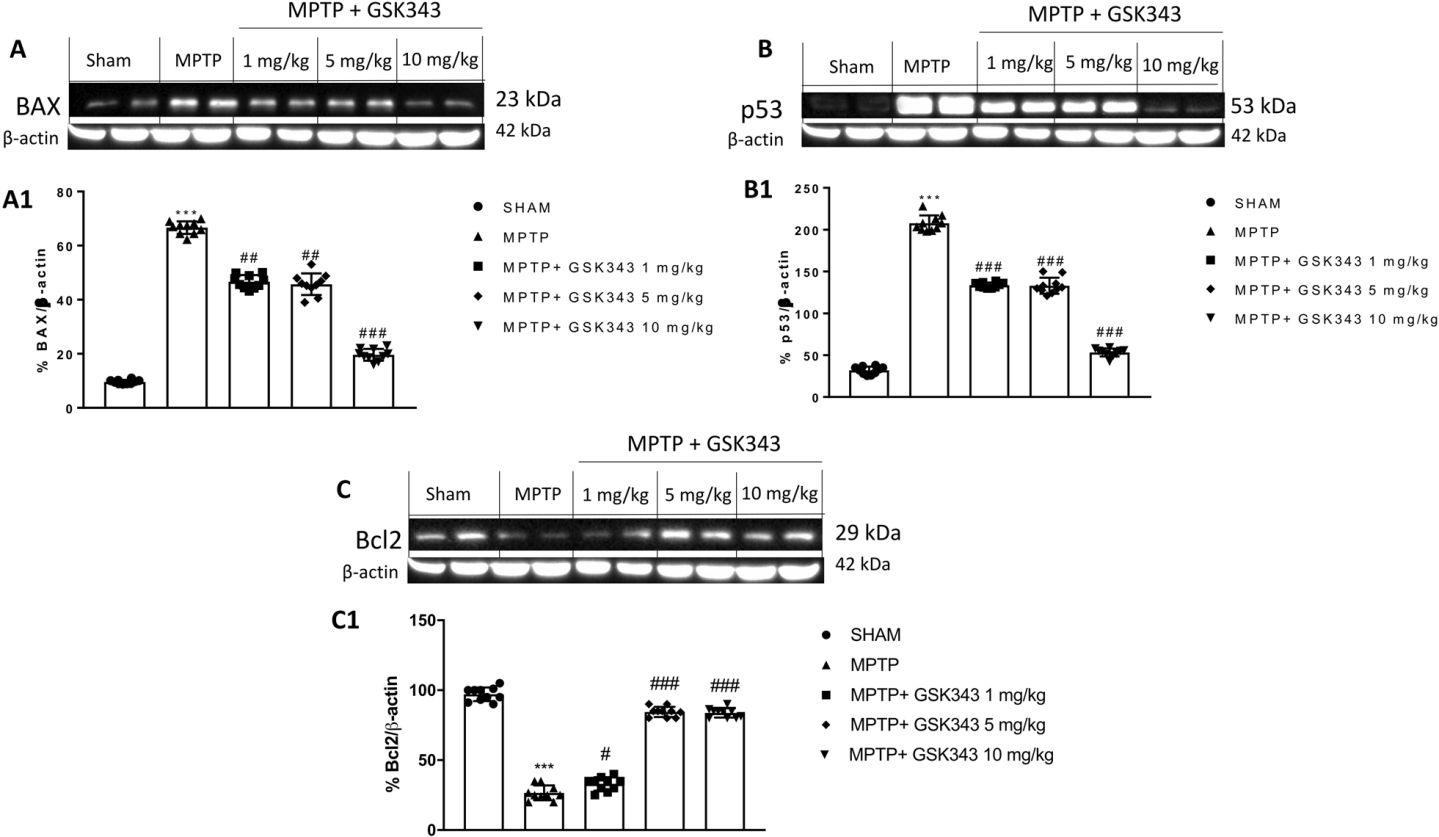
GSK-343 treatment modulated apoptosis after MPTP injection. Brain tissue of MPTP-injected mice revealed an increase of expression levels of BAX and p53, and diminished levels of Bcl-2, compared to the Sham animals. GSK-343 treatments demonstrated a significant reduction in BAX and p53 levels and increased Bcl2 levels (**A–C**; densitometric analysis **A1, B1, C1**). Data are representative of at least three independent experiments. Values are means ± SEM. One-way ANOVA test. ***p < 0.001 vs Sham; ^#^*p* < 0.05 vs MPTP; ^##^*p* < 0.01 vs MPTP; ^###^*p* < 0.001 vs MPTP

Apoptotic signal is involved in the progressive degeneration of dopaminergic neurons. Several studies have suggested that dysregulation of pro-apoptotic genes like Bax and p53 and anti-apoptotic genes like Bcl-2 play an important role in developmental DA cell death [35]. In this context, we wanted to investigate the effect of GSK-343 on pro-apoptotic proteins p-53, bax and the anti-apoptotic protein Bcl2 by western blot analysis. MPTP-injected mice showed an increased Bax expression, compared to the sham mice. GSK-343 treatment significantly reduced Bax expression at the doses of 1 mg/kg and 5 mg/kg, but a greater reduction was observed at the higher dose of 10 mg/kg (Fig. 10A, see densitometric analysis panel A1). Furthermore, western blot analysis showed that p53 levels were appreciably increased in the brain of MPTP-intoxicated mice compared to the Sham mice in which p53 is physiologically expressed. GSK-343 1 mg/kg and 5 mg/kg treatment demonstrated a significant reduction in p53 levels compared to MPTP-injected mice. In mice treated with GSK-343 at a dose of 10 mg/kg, a greater reduction in p53 expression was observed, almost comparable to sham mice (Fig. 10B, see densitometric analysis B1). Conversely, the anti-apoptotic protein Bcl-2 was significantly reduced in MPTP-injected mice compared to sham mice and GSK-343 treatment restored Bcl-2 expression to the basal levels (Fig. 10C, see densitometric analysis C1). Taken together, the results obtained demonstrated that the treatment with GSK-343 attenuates the degree of apoptosis.

## Discussion

PD is the second common neurodegenerative disease in the world [36]. It is a multi-factorial disease characterized by the degeneration of the nigrostriatal dopaminergic system. Current therapies, such as levodopa and monoamine oxidase-B (MAO-B) inhibitors, focused on ameliorating the symptoms of dopamine loss; however, alternative therapeutic strategies are needed. In the last decade, various studies focused on the role of EZH2, an essential component of PRC2, in neurological diseases [37–39]. EZH2 is a methyltransferase involved in histone methylation and transcriptional repression; it is well-known to regulate the immune system in the CNS and the balance between self-renewal and differentiation in the cerebral cortex [40]. However, emerging evidence demonstrated that EZH2 alteration or overexpression plays a key role in inflammatory diseases, such as prostatitis, colitis but also in ischemic stroke and early brain injury (EBI), revealing that its inhibition attenuates neuroinflammation and provides neuroprotective effects [41, 42]. In the field of neurodegenerative diseases, studies demonstrated an upregulation of EZH2 in the brains of PD patients, suggesting its possible pathogenic role in PD progression [11]. Therefore, based on these findings, in this paper, we aimed to evaluate for the first time the neuroprotective effects of GSK-343, a selective EZH2 inhibitor, in an in vivo model of MPTP-induced nigrostriatal degeneration. As well discussed, motor and non-motor dysfunctions are directly linked to DA degeneration which result in bradykinesia, rigidity, and tremor, typical features of PD patients [43]. Clearly, our data demonstrated a significantly decrease of motor and non-motor functions in MPTP-injected mice; however, the treatments with GSK-343 at the doses of 1, 5 and 10 mg/kg significantly ameliorated motor and non-motor functions in a dose-dependent manner, preventing the loss of locomotor agility. PD is an aging-related movement disorder mainly caused by a deficiency of neurotransmitter dopamine (DA) in the striatum of the brain and progressive degeneration of nigro-striatal DA neurons [26]. TH, tetrahydrobiopterin (BH4)-dependent and iron-containing monooxygenase, is an important enzyme which catalyzes the conversion of L-tyrosine to L-3,4-dihydroxyphenylalanine (L-DOPA), essential for biosynthesis of catecholamines including DA [28]. It has been demonstrated that TH plays a key role in the pathophysiology of PD. Therefore, in this study, we analyzed TH expression, demonstrating that MPTP-injected mice group was characterized by a significant decrease in TH expression following MPTP injection; however, the treatments with GSK-343 at doses of 1, 5 and 10 mg/kg noticeably prevented TH + expression in a dose-dependent manner, showing protective effects against MPTP-induced injury. In response to physiological demands, DAT provides neurons the ability to modulate dopamine clearance [44]. DAT promotes rapid uptake of dopamine from the extracellular space to the presynaptic neuron. The results obtained from this study showed a significant decrease in DAT expression in the substantia nigra after MPTP administration. In contrast, GSK-343 revealed a remarkable neuroprotective activity by restoring DAT levels. Furthermore, we evaluated the levels of dopamine and its metabolites such as DOPAC and HVA further confirming the effect of GSK-343 in counteracting the dramatic reduction of striatal dopamine, DOPAC and HVA levels after MPTP injection. MPTP-induced neuronal degeneration has also been linked with the redistribution of α-synuclein from its normal synaptic location to aggregates in degenerating neuronal cell bodies. Under pathological conditions, it has been demonstrated that α-synuclein progresses from monomers to inclusions through a multi-step process, which lead to the formation of soluble oligomer species that cause neuronal cell toxicity [45]. In this context, the treatments with GSK-343 demonstrated to significantly reduce α-synuclein-positive neuron number in a dose-dependent manner compared to MPTP-injected mice group, counteracting α-synuclein aggregates formation. Another important pathological feature of neurodegenerative diseases is the alteration in myelin level which is essential for various neuronal processes including impulse transmission, synaptic plasticity, and blood brain barrier integrity [46]. Accordingly, our data showed a significant myelin degradation in MPTP-injected mice, whereas it was prevented following the treatments with GSK-343 in a dose-dependent manner. There is abundant evidence documenting the relationship between histone methylation and neuroinflammation. Targeting EZH2 methyltransferase activity has been proven to be effective in multiple neuroinflammatory diseases, such as neuropathic pain, ischemic stroke and early brain injury (EBI), revealing a cross-talk between EZH2 and TRAF6/NF-κB pathway [12, 47–49]. The increase of EZH2 activity promotes the activation of TRAF6 which by degrading the inhibitory protein IκBα, activates nuclear transcription factor NF-κB contributing to the neuroinflammatory process. Based on these findings, in this study, we aimed to evaluate the effect of GSK-343 on neuroinflammation, by evaluating the TRAF6/NF-κB/IκBα pathway. In agreement with the study conducted by Yujie Luo et al. [49], our results demonstrated that EZH2 inhibition attenuated neuroinflammation via the TRAF6/ NF-κB pathway. In particular, we showed that the treatments with GSK-343 at doses of 1, 5 and 10 mg/kg were able to significantly decrease nuclear TRAF6 and NF-κB expression and restore cytosolic IκBα expression in a dose-dependent manner compared to MPTP-injected mice, counteracting inflammation. Once activated, NF-κB pathway stimulates the release of numerous pro-inflammatory mediators such as enzymes, cytokines, and chemokines, which could aggravate the disease progression [50]. Therefore, the research of anti-inflammatory therapies, aimed at modulating NF-κB pathway and its cross-talks, could represent an alternative therapeutic strategy for PD management. Accordingly, the treatments with GSK-343 showed to exert beneficial properties, reducing pro-inflammatory cytokines expression such as IL-1β, IL-2, IL-6 and IL-17A, as well as the expression of pro-inflammatory enzymes such as iNOS and COX-2 and MMPs like MMP-2 and MMP-9 in a dose-dependent manner, thus alleviating the neuroinflammatory condition. Furthermore, GSK-343 treatments demonstrated to decrease in a dose-dependent manner the expression of NIK, a kinase which plays a key role for the non-canonical NF-κB pathway activation. Clinical studies found that the damage to dopaminergic neurons is mediated by apoptosis process, mainly by p53 pathway activation [51]. Accordingly, here we identified an increase of pro-apoptotic proteins p53 and Bax, and a decrease of anti-apoptotic protein Bcl2 in the MPTP-injected mice as consequent of dopaminergic neurons damage; however, the treatments with GSK-343 in a dose-dependent manner were able to significantly decrease apoptotic process, reducing dopaminergic neurons death and confirming apoptosis modulation.

Furthermore, an incessant neuroinflammatory state can trigger the activation of glial cells in the central nervous system (CNS) such as microglia and astrocytes which can consequently promote neurodegenerative disease progression including PD [52]. Arifuzzaman et al. demonstrated that EZH2 is involved in microglial cells activation and, cell-specific knockouts of EZH2, using selective inhibitors, are able to suppress the activation of microglia [53]. Therefore, according to this, we decided to evaluate the expression of GFAP and Iba-1, markers of reactive astrocytes and microglia, respectively, showing that the treatments with GSK-343 at doses of 1, 5 and 10 mg/kg significantly decreased the levels of GFAP and Iba-1, attenuating consequently reactive astrocytes and microglia after MPTP-induced nigrostriatal degeneration. Thus, our obtained results revealed for the first time the beneficial effects of GSK-343, an EZH2 inhibitor, in a mouse model of MPTP-induced nigrostriatal degeneration, suggesting its potential therapeutic use to preserve the survival of dopaminergic neurons through neuroinflammation and apoptosis processes modulation.

## Conclusions

In conclusion, our data demonstrated for the first time that GSK-343, an EZH2 inhibitor, could be considered a promising therapeutic strategy for the management of PD thanks its abilities to counteract neuroinflammation as well as apoptosis processes in PD. However, further studies are needed to better understand the mechanism of action and the efficacy of GSK-343 in PD.

## Data Availability

All data in this study are included in this published article and its additional information files.
